# Optimal unified combination rule in application of Dempster‐Shafer theory to lung cancer radiotherapy dose response outcome analysis

**DOI:** 10.1120/jacmp.v17i1.5737

**Published:** 2016-01-08

**Authors:** Yanyan He, M. Yousuff Hussaini, Yutao U. T. Gong, Ying Xiao

**Affiliations:** ^1^ Scientific Computing and Imaging Institute, University of Utah Salt Lake City UT; ^2^ Mathematics, Florida State University Tallahassee FL; ^3^ Radiation Oncology, Thomas Jefferson University Philadelphia PA USA

**Keywords:** Dempster‐Shafer theory, evidence theory, belief and plausibility measures, dose‐volume effects, Quantitative Analyses of Normal Tissue Effects in the Clinic (QUANTEC)

## Abstract

Our previous study demonstrated the application of the Dempster‐Shafer theory of evidence to dose/volume/outcome data analysis. Specifically, it provided Yager's rule to fuse data from different institutions pertaining to radiotherapy pneumonitis versus mean lung dose. The present work is a follow‐on study that employs the optimal unified combination rule, which optimizes data similarity among independent sources. Specifically, we construct belief and plausibility functions on the lung cancer radiotherapy dose outcome datasets, and then apply the optimal unified combination rule to obtain combined belief and plausibility, which bound the probabilities of pneumonitis incidence. To estimate the incidence of pneumonitis at any value of mean lung dose, we use the Lyman‐Kutcher‐Burman (LKB) model to fit the combined belief and plausibility curves. The results show that the optimal unified combination rule yields a narrower uncertainty range (as represented by the belief–plausibility range) than Yager's rule, which is also theoretically proven.

PACS numbers: 87.55.dh, 87.55.dk

## I. INTRODUCTION

Radiotherapy, which plays an important role in the treatment of lung cancer, often leads to complications. Therefore it is important and necessary for physicians to estimate the risk of complications according to published information (available clinical data) and their experience. In 2010, the Quantitative Analysis of Normal Tissue Effects in the Clinic (QUANTEC) reviews provided focused summaries of the dose/volume/outcome information for many organs. However, uncertainties such as the measurement error of total lung volume involved in radiation therapy practice and inconsistency among the algorithms used in different institutes were ultimately reflected in the outcomes of the data analysis. QUANTEC suggested that the information in the reviews could be updated and improved with the help of new physical and statistical techniques.^1^, ^2^, ^3^ The present work introduces a statistical tool from the Dempster‐Shafer (DS) theory to evaluate dose response. Using the combination procedure in DS theory, data from multiple sources can be fused, and more specific and accurate inference may be achieved. Although there exists meta‐analysis, such as the inverse variance weighting method for data fusion, it mainly deals with the uncertain information involving randomness, and it requires unpublished results as well as published results to avoid publication bias. On the other hand, DS theory and consequently its combination rules, are capable of dealing with different types of uncertainties, and producing reasonable results based on the available information. Therefore, in the current work we focus only on the combination rules within DS theory.

We have previously implemented a case study of “belief and “plausibility” in regard to the occurrence of radiation pneumonitis (RP) as an example to demonstrate the application of the theory.^(4)^ In the current work, we discuss an optimal unified rule that provides a combined result (fused information) most similar to the individual sources of information — the lung cancer radiotherapy dose response data from different institutes.

## II. MATERIALS AND METHODS

In lung cancer radiotherapy dose response analysis, we are interested in the incidence of pneumonitis among patients who have received radiotherapy for lung cancer. The information we have (see Table 1) is the probability of pneumonitis (in the form of error bars: ± one standard deviation (SD)) at specific mean lung doses (MLD) from four institutions: Memorial Sloan‐Kettering Cancer Center^(5)^ (MSKCC), Duke University Medical Center^(6)^ (Duke), MD Anderson Cancer Center^(7)^ (MD Anderson) and the University of Michigan^(8)^ (Michigan). The incidence ranges of the pneumonitis are the same as those in the previous study,^(4)^ except that the data from Duke University are corrected as follows: We used the observed incidence of RP with respect to MLD (Table 4 from Duke^(6)^) to estimate the confidence interval [p−σ,p+σ], where p is the number of observed pneumonitis cases divided by the total number of cases n, and σ is the estimate of the standard deviation using the formula $\sqrt{p(1-p)/n}$.

In the terminology of DS theory (see Appendix A), the universal set Θ comprises the set {RP} of patients with pneumonitis after radiotherapy and the set {non‐RP} of patients without pneumonitis: Θ={RP,non−RP}. The power set $2^{\Theta }$ is defined as {Θ,{RP},{non−RP},Θ}. The propositions are in the form of “the patient belongs to A” where A is an element of the power set. We construct an m‐function over the power set to represent the uncertainty in each source of information (i.e., we assign a belief mass (a number in [0, 1]) to each proposition based on the information) indicating how strongly the information favors the proposition. For example, at MLD=8 GY from MSKCC, we assign the minimum probability 0% to the proposition of {RP} as the belief mass and also the degree of belief (i.e., m({RP})=Bel({RP})=0) and the maximum probability 6% as the degree of plausibility (i.e., Pl({RP})=6%). Then the m‐function is constructed as

(1) m({RP})=0; m({non−RP})=1−Pl({RP})=0.94; m(Θ)=Pl({RP})−Bel({RP})=0.06

Bel({RP}) may be considered as one's belief regarding the proposition {RP}, while Pl({RP}) can be considered as a measure of evidence that does not contradict the proposition {RP}. They represent the lower and upper bounds of the true probability of pneumonitis. Notice that unlike the probability measure, we have Bel({RP})+Bel({non−RP})<1. The remaining partial belief (i.e., 1−Bel({RP})−Bel({non−RP})), which is also the gap between the belief and the plausibility of pneumonitis (i.e., Pl({RP})−Bel({RP})), corresponds to the uncertainty due to the incomplete knowledge. Similarly, we construct all the m‐functions $m_{i}^{j}$ for jth MLD from ith institutions (see Appendix B).

**Table 1:** Radiation pneumonitis incidence ranges at four different dosages (Gy) from the four institutions.

	*Incidence Range of RP at Corresponding MLD*			
*Institutions*	*8 Gy*	*15 Gy*	*20 Gy*	*25 Gy*
MSKCC^5^	0%∼6%	4%∼16%	15%∼35%	—–
Duke^6^	5.76%∼14.24%	12.57%∼23.43%	10.82%∼21.18%	26.73%∼39.93%
MD Anderson^7^	—–	10%∼24%	23%∼39%	26%∼45%
Michigan^8^	0%∼7.3%	—–	—–	60%∼96%

Next, we apply a special case of Inagaki's unified rule^(9)^ — the optimal unified rule (from He and Hussaini^(10)^) based on a distance measure — to fuse the m‐functions to yield a single m‐function. The m‐function obtained (representing the combined information from the four institutions) is most similar to the individual sources of information. See Appendix A for the details of the combination rules.

## III. RESULTS & DISCUSSION

### A. The combined results from the optimal unified rule

**Figure 1:**
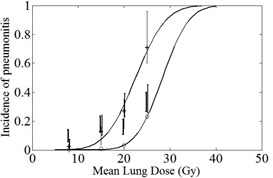
The degrees of belief (⋄) and plausibility (+) obtained from the optimal unified rule. The solid curves are the fits for the degrees of belief (lower curve) and plausibility (upper curve) using the LKB model. The error bars are from the clinical data of MSKCC,^(5)^ Duke University,^(6)^ M.D. Anderson,^(7)^ and the University of Michigan.^(8)^

The degrees of belief and plausibility of pneumonitis corresponding to the different dosages are calculated using the optimal unified rule (see Appendix C for data) and plotted in Fig. 1. The LKB model is used to fit the degrees of belief and plausibility at the four different dosages to obtain two boundary sigmoid curves.

The best estimation of the incidence of RP (at a specific dose) from the four institutions is between the degrees of belief and plausibility. For example, for a patient receiving the dosage MLD=15 Gy, the minimum incidence of radiation pneumonitis is 0.83%, while the maximum incidence of radiation pneumonitis is 12.80%. The gap between these two (the belief‐plausibility range) takes into account the uncertainty in the original information and part of the conflict among information sources. It can be interpreted as the uncertainty with which a patient belongs to either group ({RP} or {non‐RP}). Figure 1 also shows that the belief–plausibility range widens as MLD increases because the original data (see Table 1) involve more uncertainty in data from individual institutions and data conflict/inconsistency among the data at higher doses. The two boundary curves provide the belief and plausibility of {RP} continuously with respect to MLD. Figure 1 also shows that radiation pneumonitis essentially always occurs when MLD ≥; 40 Gy, and the conservative estimation of the dosage with which RP essentially always occurs is 35 Gy.

### B. Comparison of the results from the optimal unified rule to the results from DS and Yager's Rules

**Figure 2:**
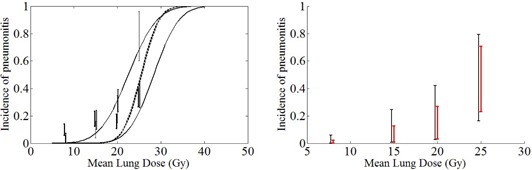
(left) The solid curves are the fits for the degrees of belief (lower curve) and plausibility (upper curve) obtained from the optimal unified rule using the LKB model. The dashed curves are the fits for the degrees of belief (lower curve) and plausibility (upper curve) obtained from Dempster's rule using the LKB model. The error bars are from the clinical data of MSKCC,^(5)^ Duke University,^(6)^ M.D. Anderson,^(7)^ and the University of Michigan.^(8)^ (right) Belief and plausibility ranges; the results from the optimal unified rule are the thicker lines in red, and the ones from Yager's rule are the thinner lines in black.

The results from DS and Yager's rules are shown in Fig. 2 and compared to the optimal unified rule.

Figure 2 (left) indicates that Dempster's rule of combination produces counterintuitive results because all the original incidence ranges from the institutions (e.g., at MLD=20 Gy) are outside the range of belief and plausibility curves; the maximum possibility of pneumonitis after combination (plausibility value) is even smaller than all the estimated minimum possibilities of pneumonitis (the lower bounds of the vertical bars) from the institutions. This result obviously is due to the renormalization (i.e., distributing the belief mass committed to the empty set to the focal elements proportionally to their belief masses), which reinforces the proposition (focal element) with a larger degree of belief. Compared to Dempster's rule, the optimal unified rule provides reasonable results. Compared to Yager's rule (see Fig. 2 (right)), the optimal unified rule produces results with smaller belief–plausibility ranges, indicating thereby relatively less uncertainty.

## Supporting information

Supplementary Material

Supplementary Material

## Appendix A. The Basics of Dempster‐Shafer Theory.

Dempster‐Shafer theory introduces measures (belief and plausibility) to model one's intuitive perception of belief/opinion and combination rules to aggregate/fuse data from multiple independent sources. Following are the basic notions of Dempster‐Shafer theory.

Let θ be the question of interest (e.g., Does a patient have radiation pneumonitis?) and let $\Theta {=\{\theta }_{1},\theta_{2},\ldots ,\theta_{n}\}$ be the collection of all possible answers (e.g., Yes and No). Θ is called the universal set or the frame of discernment. The true answer $\theta_{0}$ is unknown, and we consider the strength of support from evidence (information) for the propositions in the form of “the true answer $\theta_{0}$ is in A” where A is a subset of ΘΘ(A⊆Θ). The support of a piece of evidence for proposition A can be represented by a basic belief assignment (BBA, also called the m‐function). An m‐function assigns a number (called belief mass or simply mass) in [0, 1] to an element in the power set $2^{\Theta }$ (the collection of all the subsets of Θ):

(A1) $$m:2^{\Theta }\to [0,1]such\;that\sum_{A\subseteq \Theta }m(A)=1.$$

We further assume that no belief mass is assigned to the empty set Φ (i.e., m(Φ)=0). The element A⊆Θ is called a focal element if it has a nonzero belief mass (i.e., m(A)≠0) and the union of all the focal elements is called the core of the m‐function.

In general, the m‐function is not a traditional probability distribution function (pdf), although it is amenable to such an interpretation in a restricted sense. The m‐functions permit determination of an interval or range wherein the true probability of a proposition of interest lies. The lower bound, belief of A —Bel(A), is the sum of all m‐functions of the proper subsets B of A (B⊆A), and the upper bound, plausibility of A —Pl(A), is the sum of all the m‐functions of the subsets B that intersect A(B∩A≠Φ):

(A2) $$Bel(A)=\sum_{B\subseteq A}m(B),\;Pl(A)=\sum_{B\cap A\neq \Phi }m(B)$$

Belief is evidence supporting a proposition and it may be considered as one's weighted opinion regarding a proposition. Plausibility is evidence that does not contradict a proposition. They may be viewed as providing a lower and upper bound, respectively, on the likelihood of a proposition (being true). The gap between these two describes the uncertainty in one's belief in proposition A due to incomplete or partial knowledge.

The m‐, belief and plausibility functions in Dempster‐Shafer theory are capable of representing partial knowledge or a piece of incomplete information/evidence. In practice, different sources of evidence may provide information about the same question of interest. Assuming independence of evidence sources, Dempster‐Shafer theory introduces a combination rule to combine them to obtain a single belief for the purpose of statistical inference.

Suppose two m‐functions — $m_{1}$ with focal elements $A_{i}(1\leq i\leq n_{1})$ and $m_{2}$ with focal elements $B_{j}\;(1\;\leq \;j\;\leq \;n_{2})$ — are constructed from two distinct bodies of evidence. The conjunctive sum combines $m_{1}$ and $m_{2}$, resulting in a single function:

(A3) $$\begin{array}{l} q(C)=\sum_{A_{i}|B_{j}=C}m_{1}(A_{i})m_{2}(B_{j}),\\ q(\Phi )=\sum_{A_{i}|B_{j}=\Phi }m_{1}(A_{i})m_{2}(B_{j}), \end{array}$$

where *C* is a nonempty subset of the universal set Θ:(C⊆Θ, C≠Θ). It is quite possible that k=q(Φ)≠0 due to the fact that two bodies of evidence may support contradictory answers (we call it conflict), which violates the definition of the m‐function that be zero.

To make the resulting single function satisfy the definition of an m‐function, the Dempster's rule of combination simply ignores the conflicting evidence (discards the belief mass committed to the empty set) and inflates q(C) by multiplying them by a factor of $\frac{1}{1-k}$ such that the sum of the belief masses of the subsets is equal to unity. It is defined as an orthogonal sum:

(A4) $$m_{1}⊕m_{2}=\frac{q(C)}{1-q(\Phi )}$$

Instead of ignoring the conflict, an alternative combination rule — Yager's rule — considers the conflict part of the ignorance. Yager's rule is defined as:

(A5) $$\begin{array}{l} m^{Y}(C)=q(C),\\ m^{Y}(\Theta )=q(\Theta )+q(\Phi ), \end{array}$$

It is clear that Yager's rule reassigns the belief mass committed to the empty set to the universal set, introducing more uncertainty (see Appendix D for proof). Here, we consider an optimal unified combination rule that maximizes the similarity among the multiple datasets, thereby reducing the uncertainty range.

The unified rule produces a single m‐function for a fixed β. It is defined as follows:

(A6) $$\begin{array}{l} m_{Uni}(C)=(1+\beta q(\Phi )q(C)),\;C\subset \Theta ,C\neq \Phi ,C\neq \Theta ,\\ m_{Uni}(\Theta )=(1+\beta q(\Phi ))q(\Theta )+(1+\beta q(\Phi )-\beta )q(\Phi ),\\ m_{Uni}(\Phi )=0,\\ 0\leq \beta \leq \frac{1}{1-q(\Phi )-q(\Theta )} \end{array}$$

He and Hussaini define the optimal β such that the dissimilarity between the combined m‐function and the individual m‐functions is minimized. The dissimilarity is measured by the defined total distance (as the root mean square of the distance between the combined m‐function and $m_{i}$)

(A7) $$Tot_{Dis}=\sqrt{\frac{1}{n}(dis{\left(m_{Uni},m_{1}\right)}^{2}+dis{\left(m_{Uni},m_{2}\right)}^{2}+\ldots +dis{\left(m_{Uni},m_{n}\right)}^{2})}$$

where $dis(m_{Uni},m_{1})$ is the Jousselme's distance measure:

(A8) $$dis(m_{Uni},m_{i})=d_{Jou}(m_{Uni},m_{i})=\sqrt{\frac{1}{2}{\left({\vec{m}}_{Uni}-{\vec{m}}_{i}\right)}^{T}\underline{D}({\vec{m}}_{Uni}-{\vec{m}}_{i})},\;\underline{D}(A,B)=\frac{\left|A\cap B\right|}{\left|A\cup B\right|},$$

where $\vec{m}(\vec{m}=2^{N}\;\text{and}\;N=\left|\Theta \right|)$ is the vector form of *m*. The total distance for all four doses can be rewritten as the objective function,

(A9) $$\begin{array}{ll} J(\beta )= & \text{sqrt}(\frac{1}{2\times 3\times 4}{\left({\vec{m}}_{Uni}^{1}(\beta )-{\vec{m}}_{1}^{1}\right)}^{T}\underline{D}\left({\vec{m}}_{Uni}^{1}(\beta )-{\vec{m}}_{1}^{1}\right)+\ldots {\left({\vec{m}}_{Uni}^{1}(\beta )-{\vec{m}}_{3}^{1}\right)}^{T}\underline{D}\left({\vec{m}}_{Uni}^{1}(\beta )-{\vec{m}}_{3}^{1}\right)\\ & +\frac{1}{2\times 3\times 4}\left({\left({\vec{m}}_{Uni}^{2}(\beta )-{\vec{m}}_{1}^{2}\right)}^{T}\underline{D}({\vec{m}}_{Uni}^{2}(\beta )-{\vec{m}}_{1}^{2})+\ldots {\left({\vec{m}}_{Uni}^{2}(\beta )-{\vec{m}}_{3}^{2}\right)}^{T}\underline{D}({\vec{m}}_{Uni}^{2}(\beta )-{\vec{m}}_{3}^{2})\right)\\ & +\frac{1}{2\times 3\times 4}\left({\left({\vec{m}}_{Uni}^{3}(\beta )-{\vec{m}}_{1}^{3}\right)}^{T}\underline{D}({\vec{m}}_{Uni}^{3}(\beta )-{\vec{m}}_{1}^{3})+\ldots {\left({\vec{m}}_{Uni}^{3}(\beta )-{\vec{m}}_{3}^{3}\right)}^{T}\underline{D}({\vec{m}}_{Uni}^{2}(\beta )-{\vec{m}}_{3}^{3})\right)\\ & +\frac{1}{2\times 3\times 4}\left({\left({\vec{m}}_{Uni}^{4}(\beta )-{\vec{m}}_{1}^{4}\right)}^{T}\underline{D}({\vec{m}}_{Uni}^{4}(\beta )-{\vec{m}}_{1}^{4})+\ldots {\left({\vec{m}}_{Uni}^{4}(\beta )-{\vec{m}}_{3}^{4}\right)}^{T}\underline{D}({\vec{m}}_{Uni}^{4}(\beta )-{\vec{m}}_{3}^{4})\right)) \end{array}$$

where the combined m‐function at a specific dose ${\vec{m}}_{Uni}^{j}(1\leq j\leq 4)$ depends on the corresponding m‐functions $m_{i}^{j},m_{2}^{j},m_{3}^{j}$; the four m‐functions at all four doses share the same *β* and the optimal value of the parameter *β* satisfies $J(\beta^{*})=\underset{\beta }{\text{min}}J(\beta )$.

## Appendix B. The Constructed m‐functions at Four Different MLDs From Different Institutes.

We construct all the m‐functions on the data for a given MLD from all four institutions (see Table B.1, where each cell contains the belief masses assigned to {RP}, {non−RP} and Θ, respectively).

**Table B.1:** The m‐functions constructed based on the data from the four institutions for four dosages: the three numbers in each cell are the belief masses assigned to {RP}, {non−RP} and Θ, respectively. The constructed $m_{1}$ corresponds to institutions MSKCC and Michigan (MLD=25), $m_{2}$ corresponds to Duke, and $m_{3}$ corresponds to MD Anderson and Michigan ((MLD=8)).

	*m‐functions*								
MLD(Gy)	$m_{1}$			$m_{2}$			$m_{3}$		
8	0,	0.94,	0.06	0.0576,	0.8576,	0.0848	0,	0.927,	0.073
15	0.04,	0.84,	0.12	0.1257,	0.7657,	0.1086	0.1,	0.76,	0.14
20	0.15,	0.65,	0.2	0.1082,	0.7882,	0.1036	0.23,	0.61,	0.16
25	0.60,	0.04,	0.36	0.2673,	0.6007,	0.1320	0.26,	0.55,	0.19

## Appendix C. The Degrees of Belief and Plausibility From the Optimal Unified Rule Corresponding to Figure 1.

The degrees of belief and plausibility at different MLD from the optimal unified rule are presented in the following Table C.1, where the data correspond to Fig. 1.

**Table C.1:** The degrees of belief and plausibility.

MLD(Gy)	Bel({RP})−Pl({Rp})	Bel({non−RP})−Pl({non−RP})
8	0.03% – 2.22%	97.78% – 99.97%
15	0.83% – 12.80%	87.20% – 99.17%
20	3.22% – 26.95%	73.05% – 96.78%
25	23.05% – 70.69%	29.31% – 76.95%

## Appendix D. The Properties of the Optimal Unified Rule.

1. The optimal unified rule is applicable to combining totally conflicting sources of information where q(Φ)=1:
  Two m‐functions $m_{1}$ and $m_{2}$ are constructed on the universal set $X=\{\theta_{1},\theta_{2})$ to represent the two bodies of evidence from two totally conflicting independent sources, respectively:
  (D1) $$\begin{array}{l} m_{1}:m_{1}(\{\theta_{1}\})=1;m_{1}(\{\theta_{2}\})=0;m_{1}(X)=0,\\ m_{2}:m_{2}(\{\theta_{1}\})=0;m_{2}(\{\theta_{2}\})=1;m_{2}(X)=0, \end{array}$$
  The Dempster rule is obviously not applicable since the denominator is zero (i.e., 1−q(Φ)=0).
  Using the optimal unified rule, $\beta^{*}=0$, the combined m‐function is
  (D2) $$m(\{\theta_{1}\})=0;m(\{\theta_{2}\})=0;m(X)=1,$$
  The optimal unified combination rule rightly predicts total uncertainty or ignorance.
2. As a special case of Inagaki's unified combination rule, the present optimal unified rule introduces no greater uncertainty (i.e., narrower or equal belief‐plausibility range) than Yager's rule.
  Let ${X=\{\theta }_{1},\theta_{2})$ where $\theta_{1}$ and $\theta_{2}$ are singletons (subsets with one and only one element). The range of combined belief and plausibility using the optimal unified rule is no greater than that obtained from Yager's rule, indicating a lesser or equal amount of uncertainty introduced by the optimal unified combination rule.
  Proof: For any θ∈X,
  (D3) Pl({θ})−Bel({θ})=m(X)
  From the optimal unified rule (Eq. (A6)),
  (D4) m(X)=q(X)+q(Φ)−βq(Φ)(1−q(Φ)−q(X))≤q(X)+q(Φ)
  Since β≥0,q(Φ)≥0 and (1‐q(Φ)‐q(X))≥0,
  (D5) (1−q(Φ)−q(X))≥0;
  (D6) while from Yager's rule,we havem(X)=q(X)+q(Φ).
  Thus, the range between belief and plausibility from the optimal unified rule is less than or equal to that from Yager's rule.
3. The combined m‐function from the optimal unified rule represents the greatest amount of information similar to that represented by the individual m‐functions, that is to say, the combined m‐function (representing the fused information) remains similar to the original m‐functions (representing the original sources of information) as much as possible.
  We use the total distance ${\text{Tot}}_{\text{Dis}}$ (the root mean square of the Jousselme's distance between the combined m‐function and $m_{i}$) to measure the dissimilarity between the fused information and the original sources of information. The optimization problem we solve for β indicates that the optimal unified rule produces the combined m‐function with the most similarity.
