# Supplementary figures and images for: Optimal unified combination rule in application of Dempster‐Shafer theory to lung cancer radiotherapy dose response outcome analysis
Source: J Appl Clin Med Phys. 2016 Jan 8;17(1):4–11. doi: 10.1120/jacmp.v17i1.5737 (PMC5690231; doi:10.1120/jacmp.v17i1.5737)

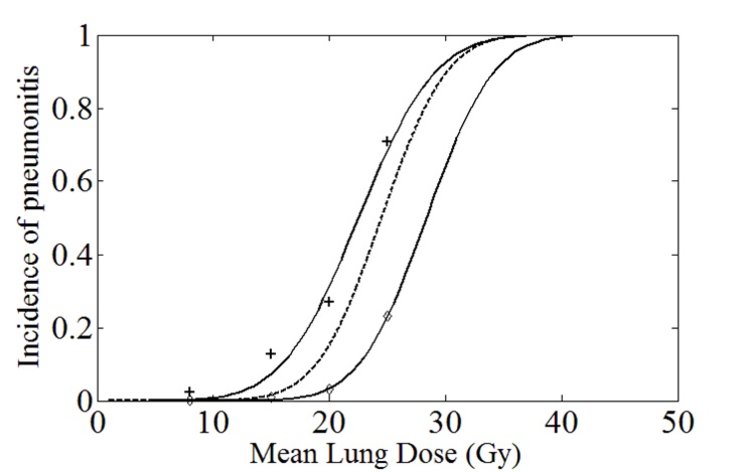

Supplement: Supplementary file 1 — Supplementary Material [file ACM2-17-4-s001.jpg]

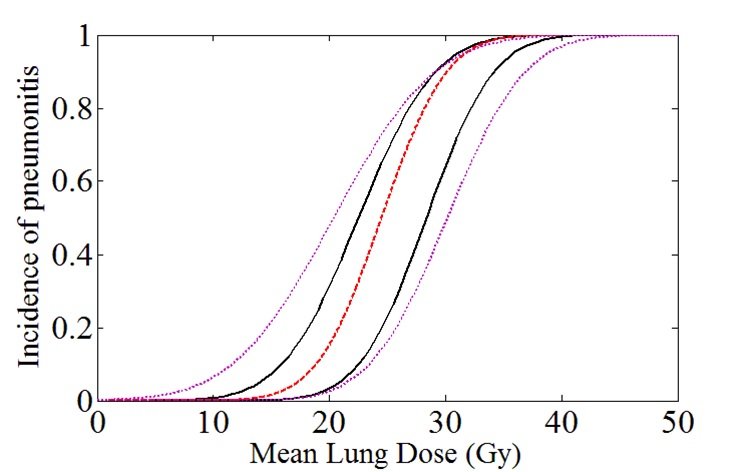

Supplement: Supplementary file 2 — Supplementary Material [file ACM2-17-4-s002.jpg]
